# Supplementary material for: Acidovorax citrulli Effector AopV Suppresses Plant Immunity and Interacts with Aromatic Dehydratase ADT6 in Watermelon
Source: Int J Mol Sci. 2022 Oct 3;23(19):11719. doi: 10.3390/ijms231911719 (PMC9570411; doi:10.3390/ijms231911719)
Supplement: Supplementary file 1 [file ijms-23-11719-s001.zip › ijms-1928028-supplementary.pdf]

Supplementary Materials

Table S1: Primers used in this study; Table S2: Bacterial strains and plasmids used in this study;

Table S1. Strains and plasmids used in this study

Strains and vectors	Characteristic	Source
Strains		
Acidovorax citrulli		
Aac5	Wild type strain, Amp^r	This lab
Δ*hrpX*	*hrpX* markerless knock-out mutant of Aac5, Amp^r	This lab
Δ*hrpG*	*hrpG* markerless knock-out mutation of Aac5, Amp^r	This lab
Δ*hrcJ*	*hrcJ* markerless knock-out mutation of Aac5, Amp^r	This lab
WT-*aopV*-cyaA	Wild-type strain Aac5 containing pBBRNolac-cyaA carrying *aopV* with its native promoter; Amp^r; Km^r	This study
Δ*hrcJ*-*aopV*-cyaA	Δ*hrcJ* containing pBBRNolac-cyaA carrying *aopV* with its native promoter; Amp^r; Km^r	This study
Escherichia coli		
DH5*α*	F-, *supE44 ΔlacU169* (*Φ80/lacZ*ΔM15), *hsd R17* , *recA1*, *endA1*, *gyrA96thi-1 relA1*	TIANGEN, Beijing, China
Agrobacterium tumefaciens		
GV3101	The wild-type strain, Rif^r	BIOMED, Beijing, China
Vectors		
pBBR1MCS-2	Broad host range expression vector with *lac* promoter, Km^r	This lab
pBBRNolac-cyaA	pBBRNolac-4FLAG containing *cyaA* gene without promoter	This lab
pBBRNolac-*aopV*-cyaA	pBBRNolac-cyaA containing *Aave_3085* gene with its native promoter	This study
pBI121	Plant expression vector containing 35S promoter, Km^r	This study
pBI121-3FLAG	pBI121 containing 3FLAG tag, Km^r	This lab
pBI121-eGFP	pBI121 containing eGFP tag, Km^r	This lab
pBI121-3FLAG-AopV	pBI121-3FLAG containing AopV	This study
pBI121-GFP-AopV	pBI121-GFP containing AopV	This study
pCAMBIA1300-nLUC	Plant expression vector containing 35S promoter, carrying nLUC tag, Km^r	Lab collection
pCAMBIA1300-cLUC	Plant expression vector containing 35S promoter, carrying cLUC tag, Km^r	Lab collection
pCAMBIA1300-nLUC-AopV	pCAMBIA 1300-cLUC fused with AopV for LCI assay, Km^r	This study

pCAMBIA1300-cLUC-ADT6	pCAMBIA1300-cLUC fused with ADT6 for LCI assay, Km^{r}	This study
pSPYNE®173	Plant expression vector containing 35S promoter, carrying nYFP tag, Km^{r}	Lab collection
pSPYCE(M)	Plant expression vector containing 35S promoter, carrying cYFP tag, Km^{r}	Lab collection
pSPYNE®173-AopV	pSPYNE®173 containing AopV for BiFC assay, Km^{r}	This study
pSPYCE(M)-ADT6	pSPYCE(M) containing ADT6 for BiFC assay, Km^{r}	This study
pGBKT7-53	Positive control bait	Lab collection
pGADT7-T	Positive control prey	Lab collection
pGBKT7-Lam	Negative control bait	Lab collection
pGBKT7-AopV	pGBKT7 containing AopV for yeast two hybrid assay, Km^{r}	This study
pGADT7-ADT6	pGADT7 containing ADT6 for yeast two hybrid assay, Amp^{r}	This study

The Km^{r}, Amp^{r} and Rif^{r} indicate resistance to kanamycin, ampicillin, and rifampicin, respectively.

Table S2. Primers used in this study

Primer name	Primer sequence (5′-3′)	Description
HB-*aopV*-F	CTAGTTCTAGAGCGGCCGCCA	For cloning the *aopV* full-length open reading frame (ORF) with its native promoter in the pBBRNolac-*aopV*-cyaA vector
HB-*aopV*-R	GGTACCGGGCCCCCCCTCGAG	
RT-*aopV*-F	CCGTCAGGCGAGTCATTTCT	For detecting *aopV* mRNA in RT-qPCR assay
RT-*aopV*-R	TTAGGGCTATGTGTTCGGCG	
121GFP-*aopV*-F	GAGCTGGACAAGGGTCTAGACGAAGATGCGGCTGTGGAA	For cloning the *aopV* full-length ORF into pBI121-eGFP vector
121GFP-*aopV*-R	TCTATCGATCAATCAGGATCCCCCCGGAGCGCGCGGCGCC	
Ac-cyaA-F	TCCCCCGGGCTGCAGGAATTCGATGGGCAGCAATCGCATCA	For cloning the *cyaA* into pBBRNolac vector to construct the pBBRNolac-cyaA vector
Ac-cyaA-R	ATCCTTGTAATCGGTAAGCTTGCTGTCATAGCCGGAATCCT	
121FlAG-*aopV*-F	GAGAACACGGGGGACTCTAGCGAAGATGCGGCTGTGGAA	For cloning the *aopV* full-length ORF in pBI121-3FLAG vector
121FLAG-*aopV*-R	GTCATCCTTGTAATCCCCGGCCCCGGAGCGCGCGGCGCC	
121FLAG-F	GGACTCTAGAGGATCCCCGGGGATTACAAGGATGAC	For inserting the 3FLAG tag into pBI121 vector to construct the pBI121-3FLAG vector
121-FLAG-R	CGATCGGGGAAATTCGAGCTCTCACTTGTCATCGTCAT	
121GFP-F	GGACTCTAGAGGATCCCCGGGATGGTGAGCAAGGGCGAG	For inserting the eGFP tag into pBI121 vector to construct the pBI121-eGFP vector
121GFP-R	CGATCGGGGAAATTCGAGCTCTCACTTGTACAGCTCGTCCATG	
RT-*rpoB*-F	GCGACAGCGTGCTCAAAGTG	Reference gene of *A. citrulli*

RT-*rpoB*-F	GCCTTCGTTGGTGCGTTTCT	strain Aac5 in RT-qPCR assay
NbAcre31-F	AATTCGGCCATCGTGATCTTGGTC	For detecting *NbAcre31* mRNA in RT-qPCR assay
NbAcre31-R	GAGAAACTGGATTGCCTGAAGGA	
NbEF1α-F	AAGGTCCAGTATGCCTGGGTGCTTGAC	Reference gene of *N. benthamiana* in RT-qPCR assay
NbEF1α-R	AAGAATTCACAGGGACAGTTCCAATACCA	
NbPti5-F	CCTCCAAGTTTGAGCTCGGATAGT	For detecting *NbPti5* mRNA in RT-qPCR assay
NbPti5-R	CCAAGAAATTCTCCATGCACTCTGTC	
AD-ADT6-F	GCCATGGAGGCCAGTGAATTCATGGACGCTCAGAAACTTCACG	For cloning the *ADT6* into pGADT7-T vector
AD-ADT6-R	CAGCTCGAGCTCGATGGATCCTCAAGCACTTTTGGGGCCAA	
nLUC-*aopV*-F	ACGGGGGACGAGCTCGGTACCGAAGATGCGGCTGTGGAA	For cloning the *aopV* into pCAMBIA1300-nLUC vector
nLUC-*aopV*-R	CGCGTACGAGATCTGGTCGACCCCGGAGCGCGCGGCGCC	
cLUC-ADT6-F	TACGCGTCCCGGGGCGGTACATGGACGCTCAGAAACTTC	For cloning the *ADT6* full-length ORF into pCAMBIA1300-cLUC vector
cLUC-ADT6-R	ACGAAAGCTCTGCAGGTCGAAGCACTTTTGGGGCCAATG	
173-AopV-F	CCCAGGCCTACTAGTGGATCCGAAGATGCGGCTGTGGAA	For cloning the *aopV* into pSPYNE®173 vector
173-AopV-R	CCCGGGAGCGGTACCCTCGACCCCGGAGCGCGCGGCGCC	
M-ADT6-F	TGGCGCGCCACTAGTGGATCATGGACGCTCAGAAACTTC	For cloning the *ADT6* into pSPYCE(M) vector
M-ADT6-R	CCCGGGAGCGGTACCCTCGAGAGCACTTTTGGGGCCAATG	
